# Smart PH-responsive drug-eluting balloons: a next-generation strategy for in-stent restenosis

**DOI:** 10.1097/MS9.0000000000004064

**Published:** 2025-10-13

**Authors:** Ayesha Ashraf, Pakeeza Saif, Mahnoor Fatima, Abdullah Imtiaz, Fahad Khan

**Affiliations:** a Department of Medicine, King Edward Medical University, Lahore, Pakistan; b Department of Medicine, Shaheed Ziaur Rahman Medical College, Rajshahi Medical University, Bangladesh; c Department of Medicine, Wah Medical College, Wah Cantt, Pakistan

*To the Editor,*

In-stent restenosis (ISR) is a major clinical issue after percutaneous coronary interventions, with reported rates of restenosis around 10%–20% for drug-eluting stents and even higher rates for bare metal stents. Inflammatory response leads to the proliferation of neointima and vascular smooth muscle at the site of the stent, leading to ISR. Other factors contributing to ISR include an acidic microenvironment created by hypoxia and inflammatory metabolism. To address this, smart drug-eluting balloons can be redesigned with coatings responding to an acidic environment and can effectively release a therapeutic payload in acidic conditions. Resultantly, drug delivery kinetics and targeting specificity can be improved^[1]^. This is in line with the TITAN Guidelines on the need for transparency in AI use in healthcare^[2]^.

Traditional drug-elluting balloons (DEBs) act by delivering anti-proliferative agents such as paclitaxel or sirolimus upon simple inflation of the balloon, thus facilitating drug release in a burst mode, which hardly coincides with the dynamic pathophysiology of ISR. Construction of pH-responsive coatings on balloon catheters can facilitate controlled drug release across the mildly acidic (i.e. pH ~ 6.0–6.8) environment present in restenotic lesions. For instance, polymers with acid-labile bonds or protonatable functional groups are used for developing coatings that only release the drug under low pH conditions. This prevents drug release to the healthy tissues and decreases the systemic exposure. Results from preclinical studies demonstrate that as much as 90% of the drug is released at pH 5.2 within 24 hours, whereas release at physiological pH 7.4 remained below 40%, a display of highly effective targeting. They prevent the common initial burst of release typical of DEBs, thus allowing drug therapy to have sustained effects during critical remodeling phases^[1,3]^.

While building next-generation smart drug-eluting balloons, following considerations are included: the drug coating needs to stay intact as it moves through the bloodstream; precise pH-triggered responses for the drug to be released in a specific acidic environment of the blocked artery; and the coating material must be biocompatible to avoid inflammatory responses or mechanical damage. New techniques like layer-by-layer polyelectrolyte deposition and bioinert hybrid materials have been shown to make these smart coatings both strong and effective enough for this procedure. These new devices must go through strict regulatory pathways in which the companies must provide strong proof that the DEBs are both safe and effective, along with the consistent drug release profiles under conditions that mimic the human body. Early clinical evidence for the safety and effectiveness of standard DEBs for ISR treatment is supported by some studies suggesting they are as effective as repeating another drug-eluting stent implantation, which paves the way for these more advanced smart-coating technologies^[4,5]^.

These advanced drug-coated balloons could be particularly useful in challenging heart procedures, such as treating blockages where arteries split into branches, narrow small blood vessels, and cases where patients need repeat treatments – all situations where doctors want to avoid using multiple stents. These “smart” DEBs could also serve as additional treatment options in blood vessels where acidity levels vary throughout the blockage, allowing for more personalized care. Future research should focus on developing balloon coatings that can respond to multiple triggers, like acidity levels and other factors that cause restenosis, such as cellular damage from oxidation. This approach would allow for more precise drug delivery and better patient outcomes^[6]^.

To conclude, the use of reliable drug-eluting balloons with pH-reactive coatings represents one of the most significant advances in the field of treating ISR: externally regulated site-specific treatment under microenvironmental conditions allows the drug to be released on site. The interdisciplinary collaboration is still highly needed to solve the manufacturing and regulatory issues, and guarantee the uptake in clinics; otherwise, there is no particular hope of transferring fancy materials science to pharmacology and clinical cardiology. It is possible to enhance the long-term vessel patency using these smart technologies and reduce their side effects; therefore, they should be immediately explored using properly designed clinical trials in order to prove their therapeutic value.

## Data Availability

Not applicable to this study.

## Research registration unique identifying number (UIN)

No human subjects were involved, as the type of research being submitted is an editorial.
